# Acquired CFTR dysfunction and dense distribution of ionocytes in nasal mucosa of children with CRS

**DOI:** 10.1007/s00405-023-07833-0

**Published:** 2023-01-20

**Authors:** Yang Han, Chao Jia, Tieshan Wang, Pengpeng Wang, Wenjing Liu, Yu Qin, Siyu Cai, Xiaojian Yang, Wei Zhang, Yuwei Liu, Xiao Xiao, Lejian He, Wentong Ge, Xin Ni

**Affiliations:** 1 Department of Otolaryngology, Head and Neck Surgery, National Center for Children’s Health, Beijing Children’s Hospital, Capital Medical University, 56 NanLishi Road, Xicheng District, Beijing, 100045 China; 2 Department of Pathology, National Center for Children’s Health, Beijing Children’s Hospital, Capital Medical University, Beijing, 100045 China; 3 Beijing Research Institute of Chinese Medicine, Beijing University of Chinese Medicine, Beijing, 100029 China; 4 Center for Clinical Epidemiology and Evidence-Based Medicine, National Center for Children’s Health, Beijing Children’s Hospital, Capital Medical University, Beijing, 100045 China; 5 Beijing Key Laboratory for Pediatric Diseases of Otolaryngology, Head and Neck Surgery, National Center for Children’s Health, Beijing Children’s Hospital, Capital Medical University, Beijing, 100045 China

**Keywords:** Nasal mucosa, Nasal polyps, Ionocytes, CFTR, Children

## Abstract

**Background:**

Ionocytes are rare cells in airway epithelium characterized by a high expression of CFTR.

**Objectives:**

To investigate the morphology and distribution of ionocytes and the function of CFTR in the nasal mucosal epithelium of children.

**Methods:**

The exfoliated cells of nasal mucosa from 101 children were detected using flow cytometry to analyze the number of ionocytes and CFTR and the difference of CFTR function. Nasal mucosa and polyps were collected from 10 children with CRSwNP. The RNAscope of FOXI1 and CFTR was detected in pathological paraffin sections. The expression and distribution of ionocytes and CFTR in nasal mucosa and polyp epithelium were observed.

**Results:**

In CRS patients, the number of ionocytes in the nasal epithelium was lower and the number of ionocytes that did not express CFTR was higher, and the function of CFTR was also decreased. The expression of CFTR in the nasal mucosa of CRS showed the characteristics of local dense distribution and increased as the inflammation expanded. The ionocytes were “tadpole-shaped” in the epithelium and gathered in the area of high CFTR expression, the intracellular CFTR was expanded in clusters. Ionocytes that did not express CFTR was more common in the nasal polyps.

**Conclusions:**

The number of ionocytes and the function of CFTR in nasal mucosa of CRS patients decreased. With the expansion of inflammation, CFTR and ionocytes showed more obvious dense distribution. Some ionocytes lost the expression of CFTR and did not show the "tadpole" shape, which may be related to the occurrence of polyps.

**Supplementary Information:**

The online version contains supplementary material available at 10.1007/s00405-023-07833-0.

## Introduction

Chronic rhinosinusitis (CRS) is a common disease characterized by chronic infection of the nasal epithelium in children, with an incidence of 2–4% [1]. Among them, most children with nasal polyps (CRSwNP) need surgical treatment, and after a long and regular drug treatment, some children with CRSwNP may still relapse, which seriously affects their quality of life. The pathogenesis of CRS is not clear, but mucosal edema and inflammatory cell infiltration can be observed under a microscope, resulting in nasal sinus orifice stenosis and obstruction of the drainage channel. This chronic inflammation breaks the dynamic balance of self-renewal, proliferation and differentiation of mucosal epithelial cells, leading to abnormal epithelial remodeling [2]. The cystic fibrosis transmembrane conductance regulator (CFTR) functions as an ATP dependent Cl^−^ channel, which is widely distributed in the mucous membrane of many organs. The CFTR distributed in the airway epithelium is critical for the normal height of the airway surface liquid and mucociliary clearance [3]. CFTR dysfunction can be divided into primary and secondary disorders. The deficiency or dysfunction of CFTR leads to the abnormal exchange of intracellular and extracellular water and Cl^−^, an increase in mucus viscosity, and a decrease in mucus clearance rate, inducing or aggravating chronic infection [4]. Due to the study of cystic fibrosis (CF) in children, primary CFTR dysfunction has been widely discussed, but there are few studies on acquired CFTR dysfunction in CRS children. Ionocytes are a newly discovered cell type in recent years, which belongs to the rare cells of the airway mucosal epithelium and has the characteristics of high expression of CFTR [5, 6]. Its biomarker is FOXI1 [7], and its main function is ion transport [8–10]. Studies have shown that after inducing and reducing the synthesis of ionocytes, the expression of CFTR in other cells increases, but the mucus clearance rate decreases [5]. Therefore, the distribution and number of ionocytes are indispensable conditions for the function of CFTR. Studies have shown that ionocytes have a proximal–distal gradient and the number of ionocytes in the nasal mucosa can reach 3%, while the proportion of ionocytes in the bronchial mucosa is less than 1% [11]. Because they are located at different levels of the respiratory tree, the ionocytes located in different areas may have different physiological functions [12]. It is suggested that ionocytes may play a more important role in the epithelium of the nasal mucosa than in the lower airway. This is a study to investigate the morphology and distribution of ionocytes and the function of CFTR in children with CRS for the pathogenesis of CRS in children.

## Methods

### Patients and groups

**Fig. 1:**
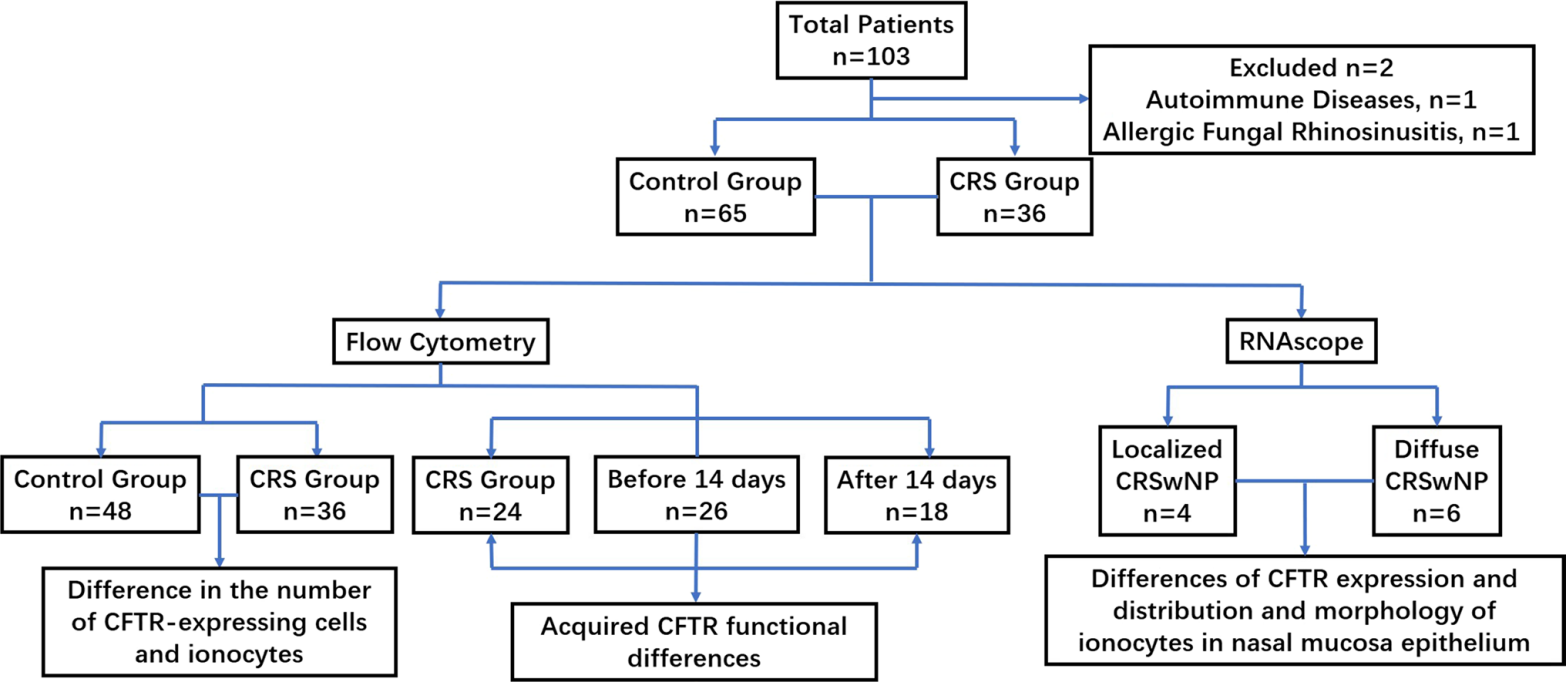
Research flowchart

This study included children who needed surgical treatment because of CRS in our Hospital from August 2020 to September 2022. Two of them were excluded because of autoimmune diseases and fungal sinusitis, and the remaining 36 patients were in the experimental group. Different from adults, the diagnosis of CRS in children mostly depends on clinical symptoms and signs, and paranasal sinus CT scan is not a necessary examination [1]. However, some young children cannot accurately describe their medical history and nasal symptoms. Therefore, to know the thickness of the nasal sinus mucosa, we selected non-CRS children who underwent surgery because of nasal bone fractures in the same period as the control group, excluding children with a Lund–Mackay score > 0 and previous history of chronic rhinosinusitis. Finally, a total of 65 children’s guardians agreed to participate in this study. All the patients in the experimental group were treated with regular drugs in the outpatient clinic for 12 weeks, but the symptoms did not significantly improve. The drugs used include: saline irrigation (250 mL twice daily), topical corticosteroids (budesonide, mometasone, or fluticasone, 2 sprays in each nostril twice daily) and mucoactive drugs. None of the patients had oral steroids. These children were treated with functional endoscopic sinus surgery. All the children in the control group were treated with closed nasal bone reduction (Fig. 1). The study was approved by the Ethics Committee of Hospital and the informed consent of all subjects was obtained (2020-Z-175).

### Flow cytometry

The exfoliated cells of the nasal mucosa from the experimental group (diseased side) and the control group (both sides) were detected using flow cytometry in 1 ml PBS buffer (C3580-0500). Centrifuge the collected cell suspension for 5 min, the supernatant was discarded, and cells were washed with PBS buffer. Fixed cells 0.5 ml Flow Cytometry Fixation Buffer (R&D Systems®, Catalog # FC004) resuscitated 5 × 10^5^ cells, incubated at room temperature for 10 min and swirled the cells continuously. The cells were washed twice with PBS buffer and centrifuged for 5 min. The cells were re-suspended with 200 μl Flow Cytometry Permeabilization/Wash Buffer (R&D Systems®, Catalog # FC005). NBP2-70747AF488 (W001-071624-AF488), PE/Cyanine7 anti-human-CD45 (304061) and NBP2-54509AF647 (1 μg) were incubated at 4 ℃ for 45 min. The cells were washed once with 1 ml Flow Cytometry Permeabilization/Wash Buffer, spun in a centrifuge for 5 min, and the supernatant was discarded. After resuspension with 400 μl Flow Cytometry Staining Buffer (R&D Systems®, Catalog # FC001), the number of ionocytes and the cells expressing CFTR in the nasal mucosa were analyzed using flow cytometry (BD FACSCantoII).

The combination of MQAE (N-(Ethoxycarbonylmethyl)-6-methoxyquinolinium bromide) dye and Cl^−^ will emit green fluorescence. The concentration of intracellular Cl^−^ was represented by fluorescence intensity. Configuration of MQAE: 1-(Ethoxycarbonylmethyl)-6-methoxyquinolinium bromide was dissolved in Krebs-HEPES buffer to prepare MQAE working solution with a final concentration of 10 mM. The cells were re-suspended in 100 μl MQAE working solution and incubated with antibody PE/Cyanine7 anti-human-CD45 and NBP2-54509AF647 (D117035) at 4 ℃ for 45 min. The cells were washed once with 1 ml Flow Cytometry Permeabilization/Wash Buffer, spun in a centrifuge for 5 min, and the supernatant was discarded. The cells were resuscitated with 400 μl Flow Cytometry Staining Buffer, and the fluorescence intensity of Cl^−^ in the exfoliated cells of the nasal mucosa was detected by flow cytometry. 

### Pathology and RNAscope

Ten patients who were diagnosed as CRSwNP in the experimental group participated in pathology and RNAscope experiments. Samples of the uncinate process nasal mucosa and polyps of 0.5 × 0.5 cm size were taken from the nasal cavity of the affected side of all subjects.

All the samples were made into paraffin sections, and then RNA in situ hybridization of CFTR and FOXI1 probes (RNAscope Probe-Hs-FOXI1, RNAscope Probe-Hs-CFTR-C2, ACD Company, USA) were carried out to detect the co-expression of CFTR and FOXI1 under a microscope. After routine dewaxing, the slices were treated with hydrogen peroxide for 10 min, targeted by repair reagent for 15 min and protease digestion for 30 min (RNAscope Multiplex Fluorescent Reagent Kit v2, ACD company, USA). The sections were then hybridized in a hybrid furnace for 7 h. After hybridization, the sections were eluted, signal amplified and stained with fluorescence. CFTR was labeled Opal 570 Reagent red fluorescence, and FOXI1 was labeled Opal 520 Reagent green fluorescence. In addition, housekeeping genes POLR2A and PPIB were used as a positive control, and bacterial gene DapB was used as a negative control. After labeling, a DAPI re-staining ScopeA1 fluorescence microscope and Isis pathological scanning system were used for image acquisition and archiving.

### Fluorescence result interpretation and cell counting

Two pathologists read the film independently and observed the fluorescence expression of all the nasal mucosa and the tissues of polyps. The three visual fields which had the largest red fluorescence signals were selected in each tissue, and 100 cells were counted continuously in each visual field. CFTR and FOXI1 RNAscope results were counted under red and green double fluorescence channels, 400×, respectively. The scores of the CFTR RNAscope results were as follows: 0 for < 1 signal, 1 for 1–4 signals, 2 for 5–9 signals, and 3 for ≥ 10 signals or clusters in each cell. At the same time, the expression of FOXI1 RNAscope in the same 100 cells was counted, and the green fluorescence expression was positive, while no expression was negative [13].

### Data analysis and statistics

The average number of cells expressing CFTR and FOXI1 in the three visual fields of each sample was calculated, and the average number of cells co-expressed by red–green fluorescence was calculated. The data were tested by rank sum test using SPSS26.0 statistical software. An independent *T* test and mean ± standard deviation was used for data with normal distribution, a Mann–Whitney *U* test was used for two groups of data that did not accord with normal distribution, a Kruskal–Wallis test was used for multiple groups of data, and the median (upper quartile and lower quartile) was used for data of multiple groups. The results were considered to be significantly different if *P* < 0.05.

## Results

### Clinical characteristics of patients

All the 36 children with CRS in this study were tested with sweat chloride levels, and the results were all under 60 mmol/l. All the children in the control group were asked for a detailed medical history to confirm that they had no history of chronic rhinosinusitis, chronic lung disease, pancreatic insufficiency or abnormal reproductive system, and no member of their family had cystic fibrosis (Tables 1, 2).

**Table 1:** Summary of clinical data

Characteristic	CRS group	Control group	*P* value
	*n* = 36	*n* = 65	
Sex (M/F)	28/8	38/27	0.051
Age (y)	10.03 ± 0.48	8.72 ± 0.42	0.054
Blood test			
Eos in blood (%)	2.49 ± 0.36	2.78 ± 0.26	0.228
Lymphocytes in blood (%)	42.15 ± 1.63	45.26 ± 1.15	0.117
Neu in blood (%)	46.9 ± 2.02	44.13 ± 1.13	0.091

Mean ± SD

**Table 2:** Clinical characteristics of patients with CRS

Characteristic	CRS
	*n* = 36
Sex (M/F)	28/8
Age (y)	10.03 ± 2.89
Medical history	
Duration of disease	29.64 ± 23.43
Allergy, *n* (%)	24 (66.66%)
Diagnosis	
CRSsNP, *n* (%)	10 (27.77%)
CRSwNP, *n* (%)	26 (72.23%)
Anatomic distribution	
Diffused, *n* (%)	9 (25%)
Localized, *n* (%)	27 (75%)
Blood test	
Eos in blood (%)	2.49 ± 2.20
Lymphocytes in blood (%)	42.15 ± 9.77
Neu in blood (%)	46.90 ± 12.14
Serum IgE (IU/ml)	148.03 ± 254.70
Imaging	
Lund–Mackay score	10.86 ± 6.45
Lund–Kennedy score	7.33 ± 2.06
Detection of exhaled nitric oxide	
FeNO50	15.00 ± 12.15
FnNO10	410.33 ± 482.66
Sweat chloride levels	41.61 ± 9.06

Mean ± SD

### The number of ionocytes in nasal mucosa of CRS decreased

**Fig. 2:**
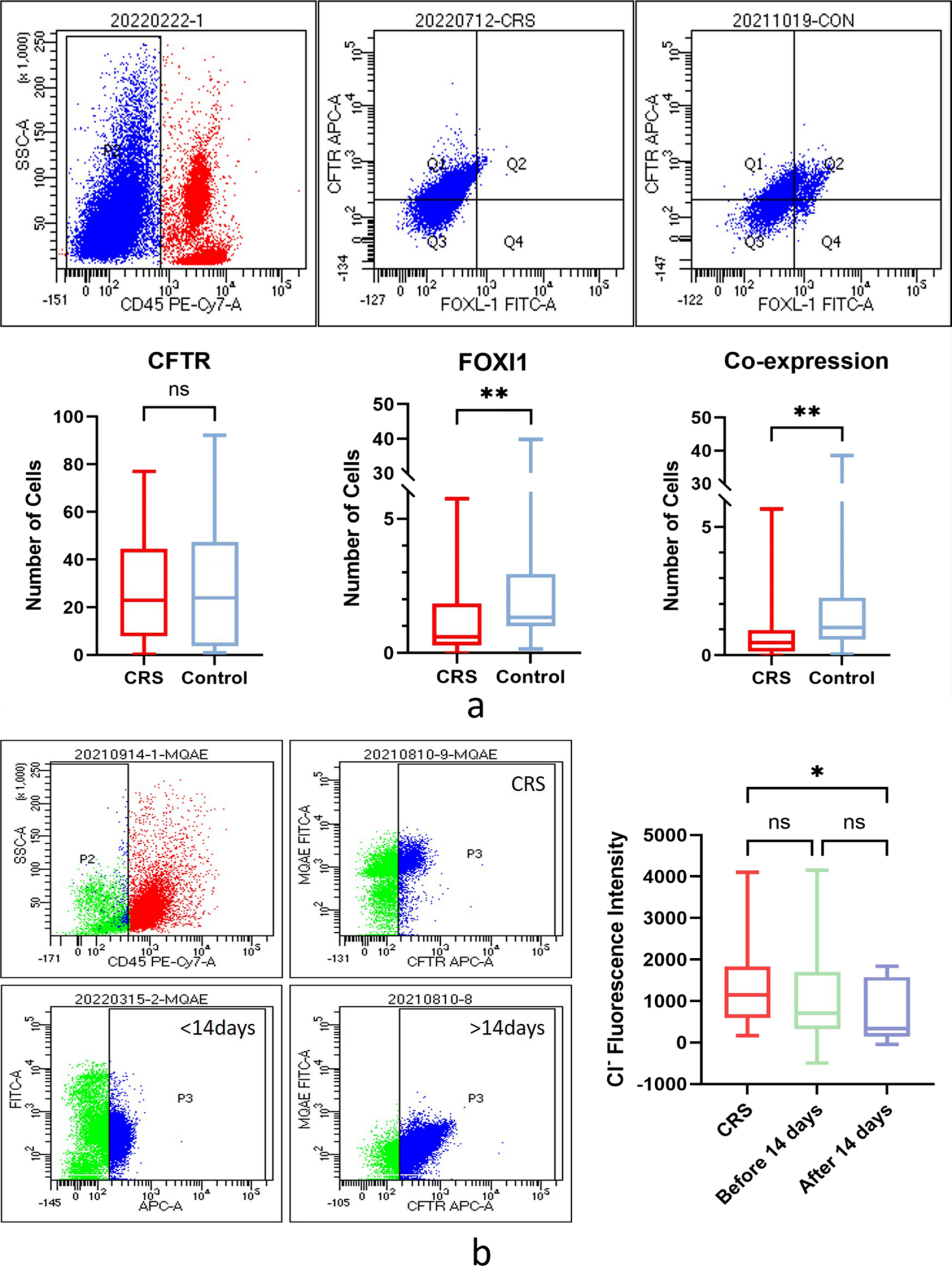
**a** To remove the inflammatory cells from the exfoliated cells in the nasal cavity, CD45-negative cells were screened for analysis. Blue represents non-inflammatory cells and red represents inflammatory cells. FOXI1 positive cells are ionocytes, Q1 is non-ionocytes that express CFTR, Q2 is ionocytes that express CFTR, and Q4 is ionocytes that do not express CFTR. Comparing the number of cells expressing CFTR and the number of ionocytes between the two groups, there was no significant difference in CFTR, but there was a significant decrease in the number of ionocytes and CFTR–FOXI1 co-expression cells in the CRS group. **b** P2 is non-inflammatory cells and P3 is CFTR-positive cells. The combination of MQAE dye and Cl^−^ will emit green fluorescence, and the fluorescence intensity represents the concentration of Cl^−^ in cells. CFTR mediates the extracellular excretion of Cl^−^, so the higher the intracellular fluorescence intensity is, the lower the CFTR function is. Comparing the intracellular fluorescence intensity among the three groups, it was found that the intracellular fluorescence intensity was the highest in the CRS group and the lowest in the nasal bone fracture over 14 day group, indicating that the CRS patients had acquired CFTR dysfunction

Comparing the number of CFTR-expressing cells and ionocytes between the two groups, there was no statistical difference in the number of CFTR, but the number of ionocytes was significantly lower in the CRS group. Comparing the ionocytes expressing CFTR between the two groups, it was found that there was a significant decrease in the expression of CFTR in the CRS group (*P* < 0.01), which means the number of ionocytes in the CRS group was lower and the number of ionocytes that did not express CFTR was higher (Table S1, Fig. 2a).

### Acquired CFTR dysfunction of the nasal mucosa epithelium in CRS

Considering the effect of nasal trauma on the function of CFTR, the control group was divided into two sub-groups according to whether the injury time was more than 14 days [14, 15], and compared with the CRS group, respectively. The combination of MQAE dye and Cl^−^ emits green fluorescence, and the fluorescence intensity represents the concentration of Cl^−^ in cells. CFTR mediates the extracellular excretion of Cl^−^, so the higher the intracellular fluorescence intensity is, the lower the CFTR function is. The results showed that the Cl^−^ transport function of CFTR in the CRS group was the lowest, and the decrease of CFTR function could also be observed in patients with nasal trauma within 14 days, while the CFTR function was the highest in patients with trauma more than 14 days prior (Table S2, Fig. 2b).

### CFTR and ionocytes showed dense distribution in the nasal epithelium of CRS group

**Fig. 3:**
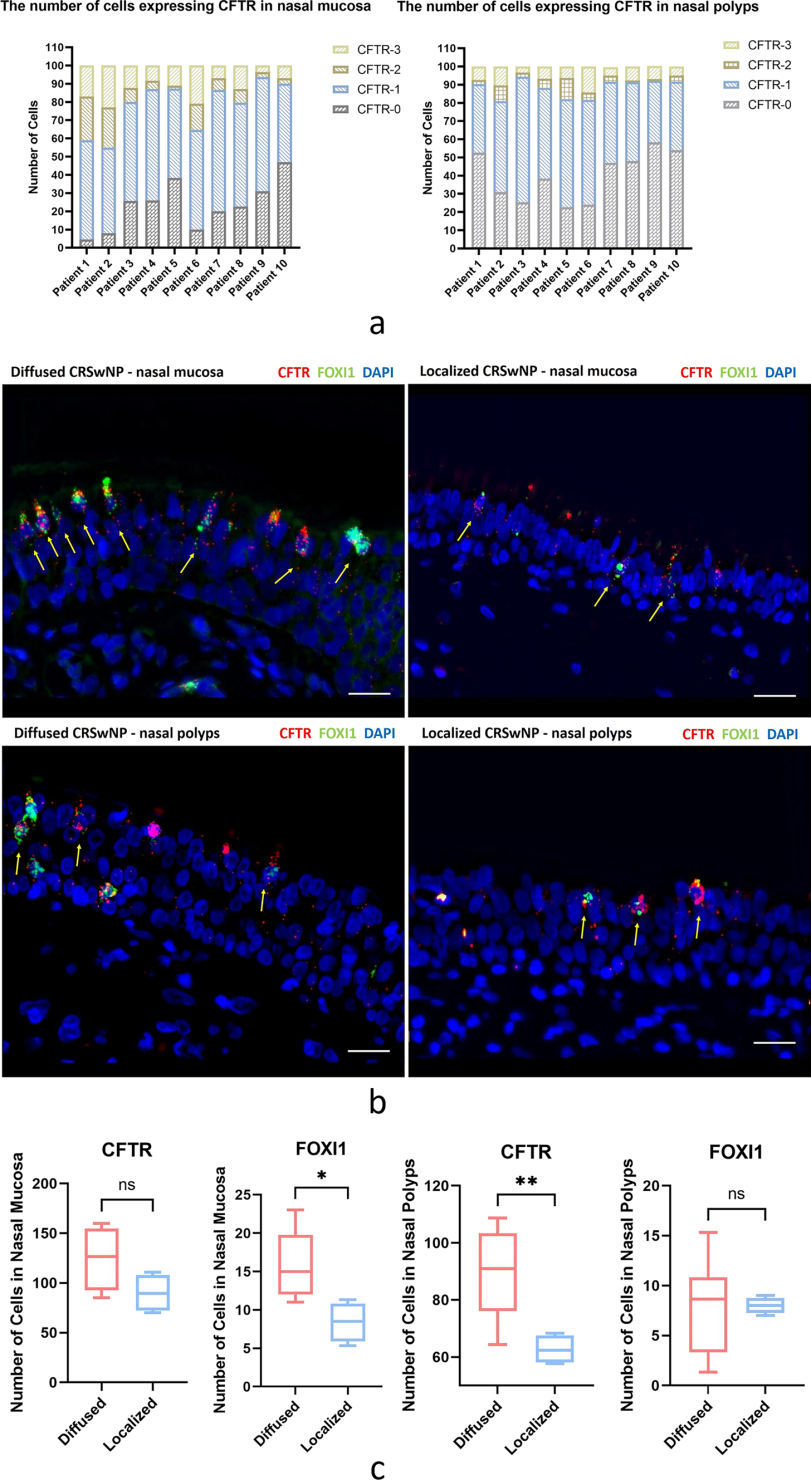
**a** Number of CFTR-expressing cells in the nasal mucosa and nasal polyp epithelium. The number of CFTR fluorescence was used to represent the expression of CFTR and scored. The number of CFTR-0 and CFTR-1 cells in the nasal mucosa and polyp epithelium was the highest, while the number of CFTR-2 and CFTR-3 cells was lower, and it was more obvious in the polyp epithelium. **b** Distribution of CFTR and ionocytes in the nasal mucosa and polyp epithelium. Scale bars = 50 μm. **c** Statistical analysis of the expression of CFTR and the number of ionocytes in the nasal epithelium. Ionocytes were denser in the mucosa of diffused CRS and CFTR was denser in polyps of diffused CRS

Under the microscope, the number of CFTR-0 and CFTR-1 cells in the nasal mucosa and polyp epithelium was the largest, while the number of CFTR-2 and CFTR-3 cells was small. This phenomenon was more prominent in the polyp epithelium (Tables S3–4, Fig. 3a). However, the expression of CFTR shows the characteristics of dense distribution. By examining co-expression of FOXI1 and CFTR to localize ionocytes, it can be seen that CFTR is highly expressed in ionocytes, and ionocytes are also clustered and distributed in areas, where CFTR expression is dense (Fig. 3b). The CRSwNP patients were divided into two sub-groups: localized and diffused CRS according to the extent of chronic inflammation of the nasal mucosa [1]. The fluorescence intensity score was used to represent the expression of CFTR in the nasal epithelium. Compared with the nasal mucosa and polyp epithelium, it was found that the expression of CFTR increased with the expansion of inflammation, especially in the polyp epithelium (*P* < 0.01). Similarly, the larger the increase of nasal mucosal inflammation, the higher the number of ionic cells (*P* < 0.05), and the denser the distribution (Table S5, Fig. 3b, c).

### Ionocytes are “tadpole-shaped” on the nasal mucosa of CRS

**Fig. 4:**
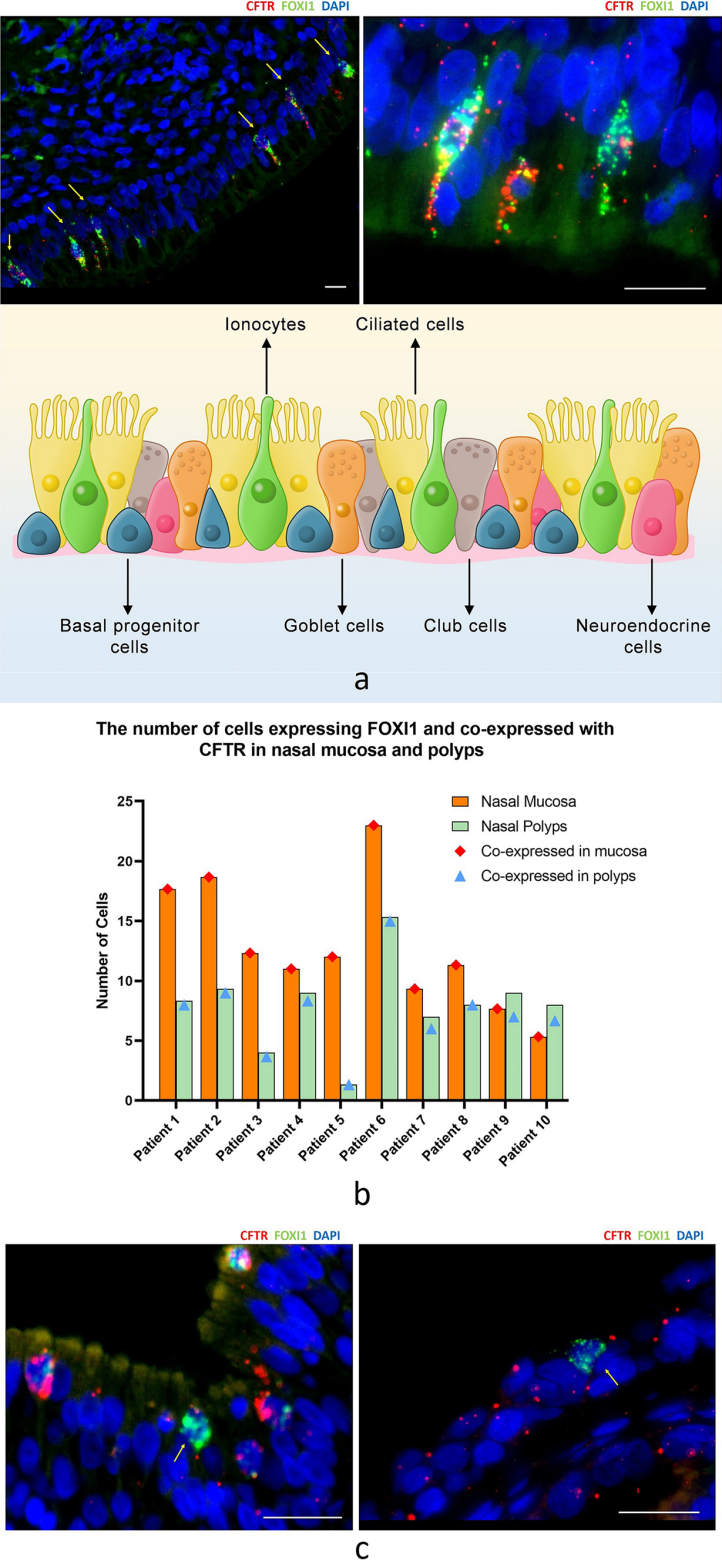
**a** Distribution of ionocytes in the nasal mucosa and the polyp epithelium was uneven and clustered between pseudostratified ciliated columnar epithelial cells. Scale bar = 25 μm; The cell is "tadpole-shaped", the tail end narrows and extends to the free edge of the ciliated cells on the epithelium surface. Scale bar = 100 μm. **b** Self-paired comparison of the number of cells co-expressed by CFTR and FOXI1 in CRSwNP patients. The histogram represents the number of ionocytes in the nasal epithelium, and the dot represents the number of CFTR–FOXI1 co-expression cells. **c** Ionocytes that do not express CFTR are more common in the polyp epithelium, and these ionocytes do not show a "tadpole" shape. Scale bar = 100 μm

Under the microscope, the distribution of ionocytes in the nasal mucosa and polyp epithelium was uneven and clustered between pseudostratified ciliated columnar epithelial cells. The cells are "tadpole-shaped", the nucleus is oval and located in the center, the head end of the cell is located at the base, and the tail end narrows and extends to the free edge of the ciliated cells on the epithelium surface (Fig. 4a).

### Ionocytes that do not express CFTR

Ionocytes that did not express CFTR could be seen in the nasal epithelium of the CRS group. In the self-paired comparison of the number of cells co-expressed by CFTR and FOXI1 in CRSwNP patients, it was found that the ionocytes that did not express CFTR in the nasal polyps were more common than those in the mucosa (Table S6, Fig. 4b). The ionocytes that did not express CFTR were also not “tadpole-shaped" (Fig. 4c).

### Distribution of ionocytes and expression of CFTR in submucosal glands

**Fig. 5:**
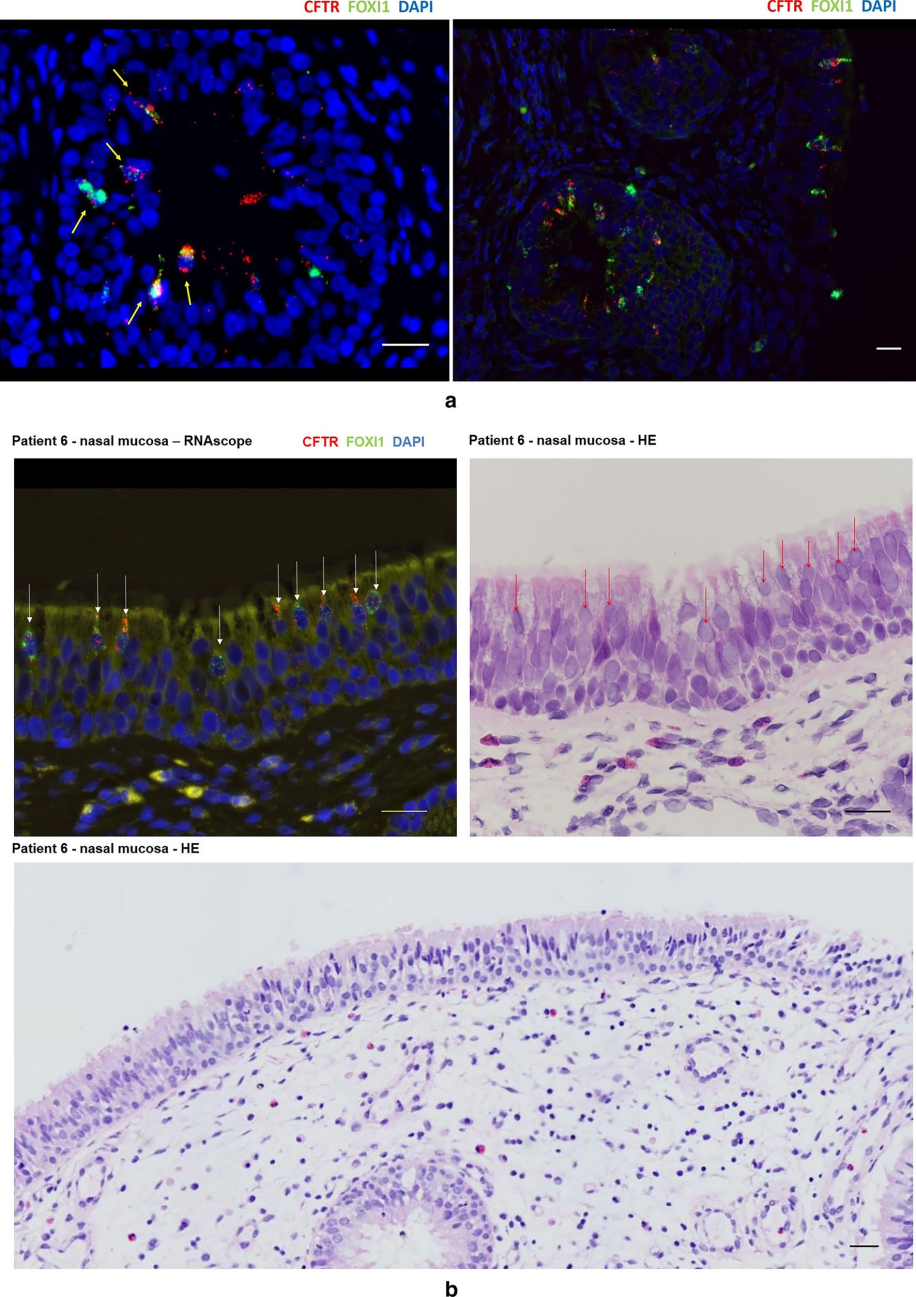
**a** Nasal mucosal epithelium with submucosal glands in CRSwNP, Scale bar = 50 μm(left); 25 μm(right). **b** Nasal mucosal epithelium of patient 6. The arrows indicate the location of ionocytes in the nasal mucosal epithelium using different staining methods. Scale bar = 50 μm. However, the infiltrating inflammatory cells in the tissue showed scattered distribution and no obvious density. Scale bar = 25 μm

The basal layer of nasal mucosa in children is thicker and the number of mucous glands is more when compared with adults. We also observed submucosal hypertrophy and proliferation of the glands in the CRSwNP patients. The distribution of ionocytes with high expression of CFTR could also be observed and the local enrichment was the same as in the nasal mucosal epithelium (Fig. 5a).

## Discussion

Ionocytes have the characteristics of high expression of CFTR [17–19], but because the proportion of ionocytes in the airway mucosa is very low, most CFTR is still expressed in other cells, such as secretory cells [20], which is consistent with our experimental results. Compared with the non-CRS group, there was no significant difference in the number of CFTR-expressing cells in the CRS group, but the number of ionocytes decreased significantly. In a study of CF and normal subjects, there was no difference in the number of ionocytes between them [11], which was inconsistent with our results. In addition to considering the age difference of the subjects, this study chose to recruit a control group from patients with nasal bone fractures. This is because, due to age and awareness, children are often unable to provide an accurate history and symptoms of rhinitis. Although the diagnosis of CRS in children does not depend on paranasal sinus CT scans, according to the CT results of nasal trauma, it is reasonable to exclude patients with a Lund–Mackay score > 0 and combined with physical examination of the children and their medical history, the non-CRS population can be selected more strictly as the control group. Not only that, but we also observed a decrease in CFTR–FOXI1 co-expression in the CRS group, indicating that, due to the stimulation of chronic inflammation, the ionocytes in the nasal mucosa lost the characteristics of CFTR expression, which means that the function of the ionocytes decreased. This decline is likely to be associated with the occurrence of nasal polyps, as this phenomenon is more common in nasal polyps.

Okuda's study described the morphology of ionocytes in the trachea. They pointed out that ionocytes are non-ciliated cells located on the surface of the respiratory mucosal epithelium and express CFTR [20] at the top, but there has been a lack of studies on the morphology of ionocytes in the nasal mucosa. Under the microscope, we observed the "tadpole” shape of the ionocytes: the tail end narrows and extends to the free edge of the ciliated cells on the epithelium surface, and CFTR is densely expressed at the tail end. This morphology makes it easier for ionocytes distributed between ciliated cells to perform the function of ion transport. However, we found that ionocytes without CFTR do not show this special shape. On the other hand, this suggests that ionocytes that do not express CFTR are likely to have lost their most important function. In addition, we also observed the local enrichment of CFTR and ionocytes in the nasal epithelium of CRS. This dense distribution can be seen in the nasal mucosa, nasal polyps and submucosal glandular epithelium [21, 22], and is more obvious with the expansion of epithelial inflammation. Interestingly, after comparing fluorescent staining with HE staining, it was found that the infiltrating inflammatory cells in the nasal mucosa and polyps did not show clusters, such as CFTR and ionocytes (Fig. 5b). This means the local high expression of CFTR cannot be explained by the severity of epithelial inflammation. We speculate that the local enrichment may suggest that this region is a synergistic functional region of highly expressed CFTR cells.

After excluding CF, acquired CFTR dysfunction was observed in patients with CRS. Considering the effect of nasal trauma on the function of CFTR [17, 23] and the recommended operation time of nasal bone fracture in children [14, 15], we divided the control group into two sub-groups. In the early stage of nasal trauma, the decrease of CFTR function can also be seen, which is related to damage of the nasal structure, mucosal laceration and changes of microenvironment caused by external force. With the repair of mucosal structure, the function of CFTR also recovered. This is significantly different from the acquired CFTR dysfunction caused by long-term chronic inflammation of nasal mucosa.

### Limitations

This is a single-center study. In real-world studies, researchers were unable to obtain nasal mucosa samples from healthy children of the same age group for a control study. Although we ensured the consistency of the sample location and made serial sections and multiple visual fields of the sample, it still cannot fully reflect the inflammation of the entire nasal mucosa and polyp epithelium due to the limitation of intraoperative sampling. In China, CF is considered to be a rare disease and there is no neonatal CF screening. Therefore, we cannot completely rule out whether there were CF patients in control group.

## Conclusions

To sum up, compared with the control group, the amount of CFTR expression in the nasal mucosa of children with CRS had no significant change, but the function was significantly decreased. With the expansion of the inflammation, the local mucosal CFTR and ionocytes showed a more obvious dense distribution suggesting that acquired CFTR dysfunction is related to chronic inflammation of the nasal mucosa. However, compared with the control group, the number of ionocytes in the nasal mucosa of CRS patients decreased significantly, and some ionocytes also lost the characteristics of expressing CFTR, and did not show the special "tadpole" shape. This phenomenon is more common in polyps, suggesting that the loss of function in ionocytes may be related to the occurrence of nasal polyps.

## Supplementary Information

Below is the link to the electronic supplementary material.

Supplementary file1 (DOCX 25 KB)

## Data Availability

We declare that submitted manuscript does not contain previously published material and is not under consideration for publication elsewhere. The manuscript is a truthful and original work without fabrication, fraud, or plagiarism. All the data and materials are available for review upon request.
