# Supplementary material for: Acquired CFTR dysfunction and dense distribution of ionocytes in nasal mucosa of children with CRS
Source: Eur Arch Otorhinolaryngol. 2023 Jan 20;280(7):3237–47. doi: 10.1007/s00405-023-07833-0 (PMC10220146; doi:10.1007/s00405-023-07833-0)
Supplement: Supplementary file 1 — Supplementary file1 (DOCX 25 KB) [file 405_2023_7833_MOESM1_ESM.docx]

| Table S1. Statistical Analysis of the Number of CFTR-expressing Cells and Ionocytes in the Nasal Mucosa Epithelium | | | |
| --- | --- | --- | --- |
| Number of cells | CRS Group | Control Group | P Value |
|  | n=36 | n=48 |  |
| CFTR | 23.08（7.85, 44.58） | 23.95（3.67, 47.38） | 0.889 |
| FOXI1 | 0.60（0.29, 1.85） | 1.32（1.00, 2.94） | 0.006** |
| Co-expression | 0.50（0.15, 0.99） | 1.07（0.61, 2.25） | 0.002** |
| Median (upper and lower quartile) | | | |

Table S2. Statistical Analysis of CFTR Function of Nasal Mucosa Epithelium

| CFTR Function | CRS Group | Control<14 Days | Control>14 Days | P Value |
| --- | --- | --- | --- | --- |
|  | n=24 | n=26 | n=18 |  |
| Cl^-^ Fluorescence Intensity | 1148.50（587.50,1832.50） | 331.75（138.75,579.88） |  | 0.497 |
|  | 1148.50（587.50,1832.50） |  | 704.25（322.63,1709.63） | 0.013** |
|  |  | 331.75（138.75,579.88） | 704.25（322.63,1709.63） | 0.296 |

Median (upper and lower quartile)

Table S3. The Average Number of Cells Expressing CFTR in the Nasal Mucosa Epithelium

| NO. | CFTR-0 | CFTR-1 | CFTR-2 | CFTR-3 |
| --- | --- | --- | --- | --- |
| Patient 1 | 4.67 | 54.33 | 24 | 17 |
| Patient 2 | 8 | 47 | 22 | 23 |
| Patient 3 | 25.67 | 54.33 | 7.67 | 12.33 |
| Patient 4 | 26 | 61 | 4.67 | 8.33 |
| Patient 5 | 38.33 | 49 | 1.67 | 11 |
| Patient 6 | 10 | 54.67 | 14.33 | 21 |
| Patient 7 | 20 | 66.67 | 6.33 | 7 |
| Patient8 | 22.67 | 57 | 7.33 | 13 |
| Patient 9 | 31 | 62.67 | 2.67 | 3.67 |
| Patient 10 | 47 | 43 | 3 | 7 |

According to the standard of CFTR score, the numbers in the table represent the average number of cells that express CFTR under three visual fields which had the largest red fluorescence signals of each sample.

Table S4. The Average Number of Cells Expressing CFTR in the Epithelium of Nasal Polyps

| NO. | CFTR-0 | CFTR-1 | CFTR-2 | CFTR-3 |
| --- | --- | --- | --- | --- |
| Patient 1 | 52.67 | 37.67 | 2.33 | 7.33 |
| Patient 2 | 31 | 50 | 8.67 | 10.33 |
| Patient 3 | 25.33 | 69 | 2.33 | 3.33 |
| Patient 4 | 38.33 | 50 | 5 | 6.67 |
| Patient 5 | 22.67 | 59.33 | 11.67 | 6.33 |
| Patient 6 | 24 | 57.67 | 4 | 14.33 |
| Patient 7 | 47 | 44.67 | 3.33 | 4.67 |
| Patient 8 | 48 | 43.33 | 1 | 7.66 |
| Patient 9 | 58.33 | 33.67 | 1 | 7.33 |
| Patient 10 | 54 | 37.67 | 3.33 | 5 |

According to the standard CFTR score, the numbers in the table represent the average number of cells that express CFTR under three visual fields which had the largest red fluorescence signals of each sample.

Table S5. Statistical Analysis of CFTR and FOXI1 Expression in the Nasal Mucosa and Polyps

| Nasal Epithelium | Diffused-CRSwNP | Localized-CRSwNP | P Value |
| --- | --- | --- | --- |
|  | n=6 | n=4 |  |
| Nasal Mucosa |  |  |  |
| CFTR | 124.50±13.26 | 90.00±9.38 | 0.067 |
| FOXI1 | 15.83±1.92 | 8.41±1.27 | 0.021* |
| Nasal Polyps |  | | |
| CFTR | 89.44±6.71 | 62.67±2.51 | 0.009** |
| FOXI1 | 7.88±1.98 | 8.00±0.41 | 0.965 |

Table S6. Self-paired Comparison of the Number of Cells Co-expression by CFTR and FOXI1 in Nasal Mucosa and Polyps

| NO. | Nasal Mucosa | |  | Nasal Polyps | |
| --- | --- | --- | --- | --- | --- |
|  | FOXI1 | Co-expression |  | FOXI1 | Co-expression |
| Patient 1 | 17.67 | 17.67 |  | 8.33 | 8 |
| Patient 2 | 18.67 | 18.67 |  | 9.33 | 9 |
| Patient 3 | 12.33 | 12.33 |  | 4 | 3.67 |
| Patient 4 | 11 | 11 |  | 9 | 8.33 |
| Patient 5 | 12.33 | 12 |  | 1.33 | 1.33 |
| Patient 6 | 23 | 23 |  | 15.33 | 15 |
| Patient 7 | 9.33 | 9.33 |  | 7 | 6 |
| Patient 8 | 11.33 | 11.33 |  | 8 | 8 |
| Patient 9 | 7.66 | 7.66 |  | 9 | 7 |
| Patient 10 | 5.33 | 5.33 |  | 8 | 6.67 |

The numbers in the table represent the average number of cells that express FOXI1 and CFTR-FOXI1 co-expression under three visual fields which had the largest red fluorescence signals of each sample.
